# Psychotic symptoms, functioning and coping in adolescents with mental illness

**DOI:** 10.1186/1471-244X-14-97

**Published:** 2014-04-01

**Authors:** Johanna TW Wigman, Nina Devlin, Ian Kelleher, Aileen Murtagh, Michelle Harley, Anne Kehoe, Carol Fitzpatrick, Mary Cannon

**Affiliations:** 1 Department of Psychiatry, Royal College of Surgeons in Ireland, Education and Research Centre Dublin, Dublin, Ireland; 2 Department of Psychiatry and Neuropsychology, School of Mental Health and Neuroscience, Maastricht University Medical Centre Maastricht, Vijverdalseweg 1, 6226 NB Maastricht, the Netherlands; 3 St Joseph’s Adolescent Unit, St Vincent’s Hospital Fairview Dublin, Dublin, Ireland; 4 Department of Clinical Psychology, Queen’s University, Belfast, Northern Ireland; 5 Department of Child and Adolescent Psychiatry, University College Dublin, Dublin, Ireland; 6 Department of Psychiatry, Beaumont Hospital Dublin, Dublin, Ireland

**Keywords:** Psychotic symptoms, Coping, Adolescents, Functioning, Mental health

## Abstract

**Background:**

Psychotic symptoms in the context of psychiatric disorders are associated with poor functional outcomes. Environmental stressors are important in the development of psychosis; however, distress may only be pathogenic when it exceeds an individual’s ability to cope with it. Therefore, one interesting factor regarding poor functional outcomes in patients with psychotic symptoms may be poor coping. This paper aimed to address the question whether 1) psychotic symptoms are associated with poorer functioning and 2) whether poor coping moderated the association.

**Methods:**

In a clinical case-clinical control study of 106 newly-referred adolescent patients with non-psychotic psychiatric disorders, coping was investigated using the Adolescents Coping Scale. Severity of impairment in socio-occupational functioning was assessed with the Children’s Global Assessment Scale.

**Results:**

Patients with non-psychotic psychiatric disorders and additional psychotic symptoms (N = 50) had poorer functioning and were more likely to use avoidance-oriented coping compared to patients with non-psychotic psychiatric disorders without psychotic symptoms (N = 56). No differences were found with respect to approach-oriented coping. When stratifying for poor/good coping, only those adolescent patients with psychotic symptoms who applied poor coping (i.e. *less* use of approach-oriented coping styles [OR 0.24, *p* < 0.015] and *more* use of avoidance-oriented coping [OR 0.23, *p* < 0.034]) had poorer functioning. However, these interactions were not significant.

**Conclusions:**

Non-adaptive coping and poorer functioning were more often present in adolescents with non-psychotic psychiatric disorders and additional psychotic symptoms. Due to small subgroups, our analyses could not give definitive conclusions about the question whether coping moderated the association between psychotic symptoms and functioning. Improvement of coping skills may form an important target for intervention that may contribute to better clinical and functional outcomes in patients with psychotic symptoms.

## Background

Environmental stressors are an important factor in the development of psychopathology [1,2]. Stress, however, has a subjective nature, which is reflected in the fact that an event becomes stressful only when it overwhelms an individual’s ability to cope with it [3]. Coping refers to the process of managing the internal and external demands created by stressful events that are considered taxing or that exceed the individual’s resources [4,5]. A large body of evidence suggests that coping impacts on both physical and mental health [5]. Coping can be described as existing along two dimensions, based on the approach or avoidance of the stressor [5]. Examples of approach-oriented coping are active problem solving, or seeking social or professional support. Examples of avoidance-oriented coping include denying or ignoring the stressor, or displaying emotional responses to the stressor such as worrying or ruminating, or engaging in wishful thinking or self-blame. In general, approach-oriented coping is seen as adaptive coping, whereas avoidance-oriented coping is usually considered as non-adaptive coping [5].

Poor coping is a well-established feature in the development of many psychiatric disorders [1,2], including the expression and development of psychosis [6-8]. Studies have for example shown that non-adaptive coping is applied more often by individuals with chronic schizophrenia as well as by individuals who experience a first psychotic episode or relapse (see for review [6]). More adaptive coping has been associated with better course and outcome; conversely, less adaptive coping has been associated with poorer course and outcome in psychosis [5,8]. Likewise, more non-adaptive coping has consistently been associated with poorer course and outcome in terms of symptom remission and general functioning [5,6]. Thus, poor coping encompasses both a high use of non-adaptive coping as well as a low use of adaptive coping. Good coping, conversely, encompasses a high use of adaptive coping and a low use of non-adaptive coping.

Recent research has shown that psychotic symptoms are prevalent in the population and commonly occur outside the range of a psychotic disorder [9]. Meta-analyses of community studies have demonstrated a median prevalence of psychotic symptoms of 5% in adults [9] and higher prevalences in children and adolescents, with a median population prevalence of 17% in 9-12 year olds and 7.5% in 13-18 year olds [10]. Furthermore, these psychotic symptoms have been suggested to be a marker of psychopathological severity in people with psychiatric disorders [11]. In both community and clinical samples of young people with non-psychotic psychiatric disorders, the presence of psychotic symptoms has been shown to be a strong marker of risk for multimorbidity (i.e. the presence of multiple co-occurring disorders) [11] and suicidal behavior [12]. In terms of etiological loading, it has furthermore been shown that a range of risk factors for psychotic disorder (including substance use, trauma and urbanicity) are more common in individuals with depressive or anxiety disorders who also report psychotic symptoms, compared to individuals with depressive or anxiety disorders who do not report psychotic symptoms [13]. With regard to treatment and treatment response, patients with major depressive disorder (MDD) who report clinical [14] or subclinical [15] psychotic symptoms have been shown to have poorer treatment outcomes than patients with MDD without psychotic symptoms.

The exact mechanisms leading to these poorer (clinical and functional) outcomes, however, remain unclear. One factor that may contribute to poor functional outcome in patients with psychotic symptoms is poor coping. For one, poor coping has been proposed to serve as a direct and strong connection between psychopathology and functioning in psychotic disorder [16]. Recent work in adolescents showed that individuals reporting subclinical psychotic experiences demonstrated more use of poor coping styles [17-20]. In a study of general population adolescents, Lin and colleagues [20] showed that the use of adaptive coping styles was associated with a decrease in psychotic experiences over time, whereas the use of non-adaptive copings styles was associated with persistence of such experiences over time. In addition to poorer coping, individuals with persisting psychotic experiences reported lower levels of functioning over time. To our knowledge, however, there has been no research to date on the association between psychotic symptoms and coping in clinical populations with (non-psychotic) psychiatric disorders, i.e. in relation to psychotic expression in the context of other psychopathology. The aim of this paper was to investigate whether poor coping might be a partial explanation for poor functional outcomes in patients with psychotic symptoms. We therefore addressed the following questions:

(i) In a clinical sample of adolescents with non-psychotic psychiatric disorders, do adolescents with additional psychotic symptoms differ in their coping styles from adolescents without additional psychotic symptoms? Specifically, do patients who report psychotic symptoms demonstrate poorer coping skills than patients who do not report psychotic symptoms?

(ii) Does coping moderate the association between psychotic symptoms and functioning?

## Methods

### Study population

The study was carried out in a large child and adolescent mental health outpatient service (CAMHS) in Ireland, which provides services to young people under 16 years living in its catchment area. The catchment area of the service has a population of approximately 380,000, of whom approximately 73,000 are under the age of sixteen years. It is divided into five sectors, each served by a multi-disciplinary team. Subjects of the study comprised all new adolescent referrals to two of these multi-disciplinary teams during the study period of 2008-2009. The area served by these two teams includes pockets of severe inner city deprivation, large suburban housing estates and more affluent areas of private housing. The study was approved by the Mater Misericordiae University Hospital’s Research Ethics Committee. Informed consent was obtained from the parents of the participants, as these were underage.

### Instruments

#### Psychotic symptoms

The interview instrument used to assess psychopathology was the Schedule for Affective Disorders and Schizophrenia for School-aged Children, Present and Lifetime versions (K-SADS-PL) [21], a well-validated semi-structured research diagnostic interview for the assessment of Axis-1 psychiatric disorders in children and adolescents. The exposure measure was the presence or absence of hallucinations and delusions. The methods used by the K-SADS to assess psychotic symptoms in children are described in detail elsewhere [22]. Briefly, auditory hallucinations and non-auditory hallucinations, including hallucinatory experiences affecting the other senses, including visual, tactile or somatic, and olfactory hallucinations were classified as psychotic symptoms. A range of delusional thoughts, including delusions of reference, control or influence, persecution, grandiosity and nihilism, were also classified as psychotic symptoms. The interviewers recorded notes of any potential psychotic phenomena during the interview. A clinical consensus meeting was held following the interviews (with IK, AM and MC) to classify these phenomena as psychotic symptoms (or not), blind to diagnoses and all other information on the participants.

#### Functioning

Severity of impairment in socio-occupational functioning was assessed with the Children’s Global Assessment Scale (CGAS), which is based on the Global Assessment Scale for Adults [23]. The CGAS is divided into ten levels, with the lowest (scored between 1 and 10) indicating very severe impairment (‘needs 24-hour care/supervision’) and the highest (scored 91 to 100) indicating a very healthy level of functioning (‘superior functioning in all areas’). CGAS scores were not normally distributed. To deal with this non-normality and to enable within-group comparisons, the CGAS score was dichotomized around the median, creating a variable indicating poorer (0) or better (1) functioning relative to the average participating patient.

#### Coping

The Adolescent Coping Scale (ACS) [24] was used to assess the coping styles that the adolescents apply. Adolescents are asked to report how frequently they use any of the 18 different coping styles described in the questionnaire on a five-point scale, ranging from ‘not at all’ (0) to ‘a great deal’ (4).

### Analyses

First, the factor structure of the ACS was investigated, because its factor structure has not been examined in clinical samples so far. This was done using parallel analysis [25], a technique that helps to determine the optimal number of factors to retain in Principal Component and Exploratory Factor Analysis. It is based on Monte Carlo simulation and is considered superior to other commonly used techniques. In parallel analysis, eigenvalues based on random data sets as well as eigenvalues based on the actual data are extracted. These random data sets are permutations of the raw data set and parallel it in number of cases and variables. Factors are retained as long as the *i*th eigenvalue extracted from the actual data is greater than the *i*th eigenvalue extracted from the random data. Next, a PCA was performed with the number of factors to extract based on the results of parallel analyses, allowing for VARIMAX rotation (i.e. orthogonal rotation, assuming uncorrelated factors).

Next, the following analyses were run to answer the research questions:

(i) In a clinical sample, do adolescents with psychotic symptoms differ in their coping from adolescents without psychotic symptoms?

In order to answer this question, two ANOVA analyses were performed, using presence of psychotic symptoms as the independent variable and (i) approach-oriented coping and (ii) avoidance-oriented coping as dependent variables. To visualize the distribution of the use of poor and good coping skills, we calculated the number of patients with (i) more and less use of approach-oriented coping and (ii) more and less use of avoidance-oriented coping separately for patients with and without psychotic symptoms by splitting around the mean adaptive and non-adaptive coping scores.

(ii) Does coping moderate the association between psychotic symptoms and functioning?

In order to answer this research question, logistic regression was performed, using presence of psychotic symptoms as independent variable and the dichotomized functioning variable as dependent variable and stratified for good and poor coping. Thus, four logistic regression analyses were run, stratified for poor coping ((i) less use of approach-oriented coping and (ii) more use of avoidance-oriented coping) and for good coping ((i) more use of approach-oriented coping and (ii) less use of avoidance-oriented coping). Stratifying for poor coping was based on the existing literature, and tested by investigating the interaction effect of coping and presence of psychotic symptoms in the model predicting functioning. All analyses were controlled for age and gender.

## Results

### Sample

The study included adolescents aged 12-16 years who were referred during the study period (2008-2009) and who could be seen in the next four weeks. This, because it was felt not to be in the best interests of adolescents and their families to expose them to a detailed research assessment, unless a clinical service could be guaranteed within a reasonable period. Of these 162 adolescents eligible for the study, 20 adolescents or their parents refused to participate. Following clinical assessment, 27 of these adolescents did not have a diagnosable psychiatric disorder. Data on the variables of interest (i.e. psychotic symptoms, coping and functioning) were available for 106 of the remaining 115 adolescents. Thus, the final data set used for the analyses consisted of 106 adolescents.

Of these 106 patients, 59 (56%) were boys and mean age was 13.8 years (SD 1.1; range 12-16). Detailed information on psychopathology has been previously reported [26]. Psychotic symptoms were reported by 50 patients (47%). There was no difference in age, gender or type of previous intervention (this last available for N = 72) between the subgroups with and without additional psychotic symptoms (all *p* > 0.05).

### Coping

**Figure 1:**
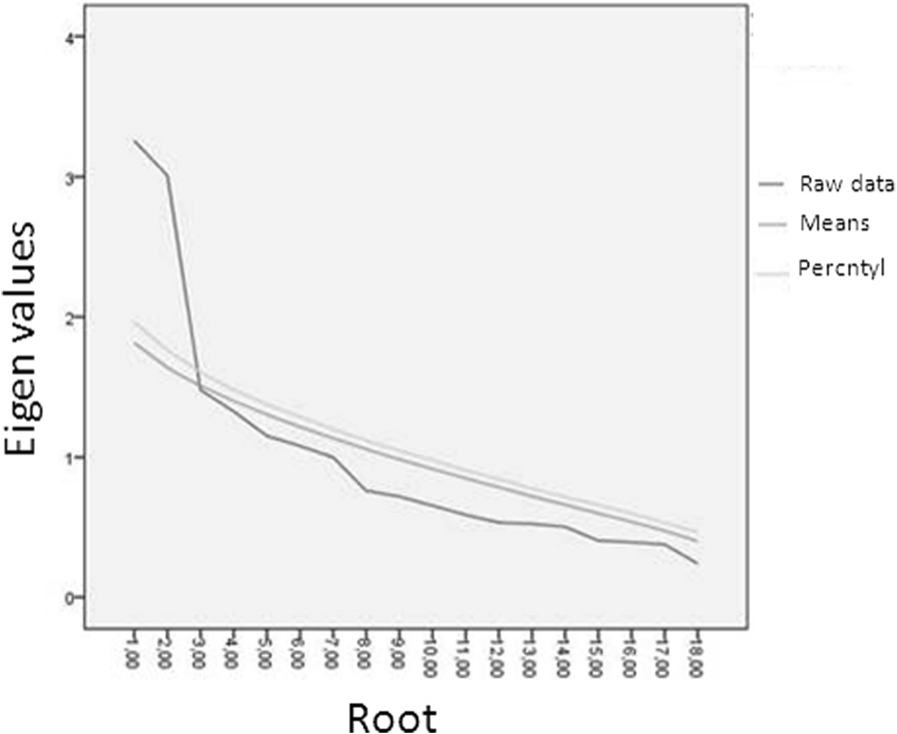
Scree plot of data-obtained eigen values of the ACS compared to randomly generated eigen values.

Results from the parallel analyses showed that the first eigenvalue of the actual data set (3.26) was higher than the eigenvalue generated from the random data set (1.81), as was the second (3.00 versus 1.64). However, the third eigenvalue generated by the actual dataset (1.48) was not higher than the one generated by the random data set (1.51) (Figure 1). Therefore, it was concluded that the ACS was best described by a two-factorial underlying structure. The distribution of the items over the two factors derived from subsequent PCA analysis and the factor loadings are shown in Table 1. Based on the content of the items, these factors were labeled “approach-oriented coping” and “avoidance-oriented” coping. Thus, two variables related to coping were constructed: (i) approach-oriented coping (sum score of all items on the ‘Approach-oriented coping’ factor) and (ii) avoidance-oriented coping (sum score of all items on the ‘Avoidance-oriented coping’ factor). Since item 5 loaded equally on both factors, this item was excluded from the calculation of the two subscales. These two variables were then split around the mean to indicate respectively less or more use of both approach-oriented and avoidance-oriented coping styles. Internal consistency of the two factors was good (Cronbach’s alpha = 0.72 for approach-oriented coping and 0.73 for avoidance-oriented coping). The two factors were not correlated (Spearman *r* = -0.06; *p* = 0.524), demonstrating that they represent dimensions of coping that exist independently.

**Table 1:** **Factor loadings of the two factors of the *Adolescent Coping Scale***

		**Approach-oriented coping**	**Avoidance-oriented coping**
1	Seek social support	0.45	
2	Solving the problem	0.51	
3	Work hard to achieve	0.40	
4	Worry		0.63
5	Spend time with boyfriend/girlfriend	0.19	0.19
6	Social action	0.61	
7	Wishful thinking		0.64
8	Not coping		0.70
9	Tension reduction		0.44
10	Seek to belong		0.50
11	Ignore the problem		0.29
12	Self-blame		0.67
13	Keep to self		0.41
14	Seek spiritual support		0.45
15	Focus on the positive	0.71	
16	Seek professional help	0.50	
17	Seek relaxing diversions	0.74	
18	Physical recreation	0.63	
	Mean factor loading	0.57	0.52

NB Item 5 loaded equally on both factors; therefore, this item was excluded from calculating the subscales and the mean factor loadings.

### Coping and psychotic symptoms

There was no difference in use of approach-oriented coping between patients with (mean score 23.6, SD 6.0) and without (mean score 24.1, SD 5.6) psychotic symptoms (F(1,99) = 0.01; *p* = 0.926). Patients with psychotic symptoms used more avoidance-oriented coping styles (mean score 27.0, SD 6.1) than patients without psychotic symptoms (21.7, SD 6.5) (F(1,99) = 15.50; *p* < 0.001). Using the dichotomized coping variables, the distribution of less/more use of good and poor coping across patients with and without psychotic symptoms is shown in Table 2.

**Table 2:** The distribution of good and poor coping over patients with and without psychotic symptoms

	**Avoidance-oriented coping**		**Approach-oriented coping**	
	**Patients without psychotic symptoms**	**Patients with psychotic symptoms**	**Patients without psychotic symptoms**	**Patients with psychotic symptoms**
Less use	39 (70%)	22 (44%)	27 (54%)	29 (58%)
More use	17 (30%)	28 (56%)	29 (46%)	21 (42%)
*Total*	*56*	*50*	*56*	*50*
	*χ*^*2*^(1) = 7.11; *p* < 0.008		*χ*^*2*^(1) = 1.02; *p <* 0.314	

### Functioning and psychotic symptoms

The proportion of patients with lower functioning was significantly higher in the group of patients with additional psychotic symptoms compared to the group of patients without psychotic symptoms (*χ*^*2*^(1) = 9.69; *p* < 0.002) (Table 3).

**Table 3:** The distribution of lower and higher functioning over patients with and without psychotic symptoms

	**Patients without psychotic symptoms**	**Patients with psychotic symptoms**
Lower functioning	20 (36%)	33 (66%)
Higher functioning	36 (64%)	17 (33%)
*Total*	*56*	*50*
	*χ*^*2*^(1) = 9.69; *p* < 0.002	

### Coping as a moderator of the association between psychotic symptoms and functioning

In Table 4, the odds ratios (ORs) of the stratified analyses are depicted that show that patients with psychotic symptoms have lower levels of functioning compared to patients without psychotic symptoms, but that this association is stronger (and significant only) for patients with psychotic symptoms who apply poor coping (i.e. *less* approach-oriented and *more* avoidance-oriented coping) compared to patients who apply good coping (i.e. *more* approach-oriented and *less* avoidance-oriented coping). However, when testing for significance of the moderation effects (i.e. the interaction between presence of psychotic symptoms and poor coping), no significant interactions were found for approach-oriented coping (Z = -0.80, *p* = 0.422) or avoidance-oriented coping (Z = -1.51, *p* = 0.132).

**Table 4:** Psychotic symptoms predicting functioning stratified for good and poor coping

	**Patients without psychotic symptoms (N = 56)**	**Patients with psychotic symptoms (N = 50)**
		**OR 95% CI p-value**
Good coping		
Less use of avoidance-oriented coping	a	0.47 0.16, 1.37 0.165
More use of approach-oriented coping	a	0.41 0.13, 1.33 0.137
Poor coping		
More use of avoidance-oriented coping	a	**0.23** 0.06, 0.90 0.034
Less use of approach-oriented coping	a	**0.24** 0.08, 0.75 0.015

a: reference category. ORs given in bold are significant at α=0.05.

## Discussion

The current study showed that, in a clinical sample of adolescents referred to mental health services, the presence of psychotic symptoms in adolescents with non-psychotic psychiatric disorders was associated with both lower levels of functioning and more use of avoidance-oriented coping styles compared to adolescents with psychiatric disorders without psychotic symptoms. Although no significant moderation effects were found, probably due to too small subgroups and thus limited statistical power, the stratified analyses (stratified for good or poor coping) suggested that individuals with psychotic symptoms only had lower levels of functioning if they used poor coping (i.e. *less* use of approach-oriented coping styles and *more* use of avoidance-oriented coping styles). The findings of the current paper suggest that psychotic symptoms, poor coping and poor functioning are associated, but they cannot give definitive conclusions on the question whether poor coping moderates the association between psychotic symptoms and poor functional outcomes.

The Adolescent Coping Scale was found to be best represented by two underlying factors in the current sample, reflecting approach-oriented (adaptive) and avoidance-oriented (non-adaptive) coping. In line with earlier work on coping [2,5], these factors were shown to be independent, though not mutually exclusive, dimensions of coping. This independence was supported by the facts that the two factors were not correlated. Also, both *less* use of approach-oriented coping and *more* use of avoidance-oriented coping were suggestively associated with lower levels of functioning in patients with psychotic symptoms. Literature on coping in mental health research usually focuses mostly on avoidance-oriented or other non-adaptive coping styles. The current study, however, underlines the importance of also including adaptive coping, such as approach-oriented coping, in relation to mental health research, as suggested by Roe and colleagues [8] who stress the importance of resilience in the context of mental health.

As we hypothesized, and consistent with complementary research [5,6,8], patients with psychotic symptoms applied more avoidance-oriented coping than patients without psychotic symptoms. However, no differences were found with regard to approach-oriented coping; this is somewhat surprising since earlier work in the context of psychosis has shown that more adaptive coping is associated with better outcome [8]. Patients with psychiatric disorders and psychotic symptoms did, however, report less use of approach-oriented coping, i.e. the (small) difference was in the expected direction, although non-significant.

Patients with psychiatric disorders and psychotic symptoms were rated with lower levels of functioning compared to patients with psychiatric disorders but without psychotic symptoms. Level of daily functioning is an important outcome in psychopathological research in addition to clinical outcome, especially from the point of view of the patient and their social context, clinicians and society [27], and provides important information regarding an individual’s situation that is not per definition the same as or dependent on clinical diagnosis [28,29]. Thus, the current study underlines the importance of functioning as an outcome of interest in the context of psychotic symptoms, especially regarding possible avenues for intervention involving, for example, coping.

There are several possible explanations for the finding that adolescents with psychiatric disorders and additional psychotic symptoms report poorer functioning. It has been suggested that psychotic symptoms can be seen as an “index of severity” of psychopathology [11]. Thus, individuals with psychotic symptoms may be more ill: individuals with psychiatric disorders who report psychotic symptoms simply have more symptoms of psychopathology than individuals with psychiatric disorders who do not report psychotic symptoms. To make sure that it is not the more severe psychopathological loading that explains the association between psychotic symptoms and functioning, we ran a post-hoc analysis in which presence of psychotic symptoms predicts functioning, while controlling for number of mental disorders present (as an index of psychopathological severity). After controlling for this, the effect of psychotic symptoms diminished somewhat but remained significant, showing that the effect of psychotic symptoms on functioning can be partly, but not wholly, explained by severity of illness (data not shown). Earlier work has also shown that common mental disorders with and without additional psychotic symptoms differed quantitatively by indicators of severity, course, onset, and environmental and familial risks, indicating that the co-presence of psychotic symptoms in non-psychotic psychiatric disorders is a common and functionally and etiologically highly relevant feature [13]. Another explanation may be that individuals with additional psychotic symptoms represent a subgroup of adolescents in which psychotic symptoms are a relatively late expression of a developmental pathway that has started earlier in life. It has been suggested that whereas psychotic symptoms may arise in relatively later stages of psychopathological development, other issues such as cognitive impairments and poor social functioning may be present already in relatively early phases [30]. Since these domains have been shown to be predictive of later functioning [28,31,32], it may be that the individuals with psychiatric disorders with additional psychotic symptoms represent a subgroup of adolescents in which psychotic symptoms are an expression of such impairments.

Individuals with psychiatric disorders and additional psychotic symptoms reported both poorer coping and poorer functional outcome. This, in combination with the suggestive moderation effect of coping, suggests that individuals with psychotic symptoms may be less able to handle stressful situations and that this may affect their functioning in daily life. This is especially problematic since many studies have demonstrated strong associations between stress and/or trauma and psychotic development. These associations can be bidirectional. A large body of literature has shown that trauma can lead to psychotic development [33,34]. Vice versa, many studies have reported that stressful situations such as trauma [34-36] and stressful life events [37,38] are more prevalent in individuals with psychotic symptoms and that stressful events often precede exacerbation of psychotic symptomatology [38]. In fact, exposure to early adversities has been shown to sensitize an individual to pathogenic effects of later stressful life events in the context of psychosis [39]. However, other studies have reported *fewer* stressful life events in individuals with recent-onset schizophrenia [40] and *fewer* daily hassles in individuals at Ultra High Risk (UHR) for psychosis [41], although these individuals did perceive the adverse events that they encountered as less controllable, less well handled and more distressing. A study by Docherty and colleagues [37] furthermore showed that life events only lead to increases in symptom level in those patients who were most emotionally reactive to stress. Thus, it seems that a heightened sensitivity to stress may be the driving force in the pathogenic effect of environmental stress. This is in line with a large body of literature that has shown that individuals who are liable to psychosis are thought to be more sensitive to stress [42]: patients with psychotic disorder as well as their healthy siblings [43,44], individuals considered at UHR for psychosis [41], individuals with schizotypal personality disorder [38] and individuals from the general population at heightened psychometric risk for psychosis [45] all have been shown to be more reactive to stressful events.

### Limitations

The findings of the current study should be interpreted in light of its strengths and limitations. Important strengths include the thorough assessment of psychiatric disorders, using a reliable, valid and widely used diagnostic interview conducted by highly trained professionals, the relatively large sample size for an in-depth interview study, and the clinical case-clinical control design. Also, the study focused not only on non-adaptive coping, as is the case with most research on coping, but also incorporated adaptive coping. In this way, it underlined the relative nature of coping, by showing that it is not so much the yes/no application of certain coping styles, but the degree to which one applies good or poor coping. Inevitably, the use of clinical in-depth interviews limits the use of extensive samples; as a result, the subgroup analyses involved smaller groups and, because of this, confidence intervals are wide in some cases. This limited statistical power may well explain why no significant moderation effects were found and replication of the study is therefore warranted. Another limitation is that since the analyses pertained to cross-sectional data, no conclusions regarding causality or directionality of the effects can be drawn. Use of illicit drugs that may be associated with the presence of psychotic symptoms was not taken into account in the current paper. Last, since only those patients were included who could be offered clinical service within 4 weeks of referral, the current sample may have relatively high levels of psychopathology. However, this ‘enrichment’ for psychopathological severity enabled us to test the association between the psychotic symptoms and functional outcome with maximal statistical power. Future research may address the development of coping and its moderating role between psychotic symptoms and functional outcome over time, ideally in the context of a larger intervention study.

### Clinical implications

We have demonstrated that individuals with psychotic symptoms have poorer coping skills, which may make them less capable of managing these stressors in a healthy and effective way. Thus, stress seems to be, at least partly, in the eye of the beholder; this has important clinical implications with regard to our current findings, as our results suggestively show that having both psychotic symptoms and poorer coping leads to poorer functioning. Intervention strategies aimed at improving coping skills could offer important possibilities for attenuating this association, and thus potentially improving functional outcome. Coping has been shown to be modifiable through stress management [5] and, more specifically in the context of psychosis, psychosocial interventions such as cognitive behavioral therapy (CBT) have shown potential in modifying coping in individuals with psychotic symptoms [46-48]. When looking more in-depth at the specific coping items of the ACS, it can be seen that the items loading highest on the avoidance-oriented coping factor were ‘not coping’, ‘self-blame’, ‘wishful thinking’ and ‘worry’. The finding that avoidance-oriented coping is strongly expressed through these particular types of coping styles suggests that such feelings, cognitions and actions may deserve extra attention when offering clinical help. Similarly, items that loaded the highest on the approach-oriented coping factor were ‘seek relaxing diversions’, ‘focus on the positive’ and ‘physical recreation’. Therefore, such measures are not merely common sense advice, but represent strong expressions of approach-oriented coping and thus, their importance should be underlined when discussing potential improvements regarding coping. Encouraging patients to apply these easy-to-use strategies may form an accessible and easy route for better coping and, in turn, better functioning.

When addressing the concept of coping for assessment, intervention or research purposes, it should be kept in mind that coping is a very dynamic concept. Not only may coping styles and skills change dramatically during adolescence [20,49], coping styles and skills have also been shown to change and develop in the course of disease development and recovery [8]. Furthermore, coping is assumed to show large individual differences based on factors such as personality, individual history and social context [2,50]. However, this dynamic nature is, in fact, advantageous in that it suggests that individuals can actively influence its development; this in turn may enhance feelings of control and empowerment that are vital for healthy recovery in individuals with psychosis [8,51].

## Conclusions

Non-adaptive coping and poorer functioning were more often present in adolescents with non-psychotic psychiatric disorders and additional psychotic symptoms. Due to small subgroups, our analyses could not give definitive conclusions about the question whether coping moderated the association between psychotic symptoms and functioning. Improvement of coping skills may form an important target for intervention that may contribute to better clinical and functional outcomes in patients with psychotic symptoms.

## Competing interests

The authors declare that they have no competing interests.

## Authors’ contributions

JW performed the analyses and wrote the manuscript. ND participated in the design and coordination of the study and helped to draft the manuscript. IK contributed to the analyses and writing of the manuscript. AM and AK participated in the coordination and data collection of the study and drafting of the manuscript. MH helped with the drafting of the manuscript. CF and MC conceived of the study, participated in its design and coordination and helped to draft the manuscript. All authors read and approved the final manuscript.

## Pre-publication history

The pre-publication history for this paper can be accessed here:

http://www.biomedcentral.com/1471-244X/14/97/prepub

## References

[B1] Gunnar MR Vaszquez D Stress neurobiology and developmental psychopathology. Developmental psychopathology Developmental Neuroscience 2006 II Second Hoboken, New Jersey: Wiley & Sons, Inc 533 577

[B2] Kessler RC Price RH Wortman CB Social factors in psychopathology: stress, social support, and coping processes Ann Rev Psychol 1985 36 1 531 572 10.1146/annurev.ps.36.020185.002531 3883893

[B3] Gunnar MR Quevedo K The neurobiology of stress and development Annu Rev Psychol 2007 58 145 173 10.1146/annurev.psych.58.110405.085605 16903808

[B4] Lazarus RS Folkman S Stress, appraisal, and coping 1984 New York: Springer Publishing Company

[B5] Taylor SE Stanton AL Coping resources, coping processes, and mental health Annu Rev Clin Psychol 2007 3 377 401 10.1146/annurev.clinpsy.3.022806.091520 17716061

[B6] Phillips LJ Francey SM Edwards J McMurray N Strategies used by psychotic individuals to cope with life stress and symptoms of illness: a systematic review Anxiety Stress Copin 2009 22 4 371 410 10.1080/10615800902811065 19333796

[B7] Ritsner MS Ratner Y The long-term changes in coping strategies in schizophrenia: temporal coping types J Nerv Ment Dis 2006 194 4 261 267 10.1097/01.nmd.0000207361.81947.52 16614547

[B8] Roe D Yanos PT Lysaker PH Coping with psychosis: an integrative developmental framework J Nerv Ment Dis 2006 194 12 917 924 10.1097/01.nmd.0000249108.61185.d3 17164630 PMC1790964

[B9] Van Os J Linscott R Myin-Germeys I Delespaul P Krabbendam L A systematic review and meta-analysis of the psychosis continuum: evidence for a psychosis proneness-persistence-impairment model of psychotic disorder Psychol Med 2009 39 2 179 195 10.1017/S0033291708003814 18606047

[B10] Kelleher I Connor D Clarke MC Devlin N Harley M Cannon M Prevalence of psychotic symptoms in childhood and adolescence: a systematic review and meta-analysis of population-based studies Psychol Med 2012 1 1 1 7 10.1017/S0033291711002960 22225730

[B11] Kelleher I Keeley H Corcoran P Lynch F Fitzpatrick C Devlin N Molloy C Roddy S Clarke MC Harley M Arseneault L Wasserman C Carli V Sarchiapone M Hoven C Wasserman D Cannon M Clinicopathological significance of psychotic experiences in non-psychotic young people: evidence from four population-based studies Br J Psychiat 2012 201 1 26 32 10.1192/bjp.bp.111.101543 22500011

[B12] Kelleher I Lynch F Harley M Molloy C Roddy S Fitzpatrick C Cannon M Psychotic symptoms in adolescence index risk for suicidal behaviour: findings from two population-based case–control clinical interview studies Arch Gen Psychiatry 2012 69 12 1277 1283 10.1001/archgenpsychiatry.2012.164 23108974

[B13] Wigman JTW van Nierop M Vollebergh WAM Lieb R Beesdo-Baum K Wittchen HU van Os J Evidence that psychotic symptoms are prevalent in disorders of anxiety and depression, impacting on illness onset, risk, and severity—implications for diagnosis and ultra–high risk research Schizophr Bull 2012 38 2 247 257 10.1093/schbul/sbr196 22258882 PMC3283146

[B14] Perlis RH Uher R Ostacher M Goldberg JF Trivedi MH Rush AJ Fava M Association between bipolar spectrum features and treatment outcomes in outpatients with major depressive disorder Arch Gen Psychiat 2011 68 4 351 21135313 10.1001/archgenpsychiatry.2010.179 PMC3794668

[B15] Wigman JTW van Os J Abidi L Huibers MJH Roelofs J Arntz A Kelleher I Peeters FPML Subclinical psychotic experiences and bipolar spectrum features in major depressive disorder: association with outcome of psychotherapy. Psychol Med 2013 doi:10.1017/S0033291713000871 10.1017/S0033291713000871 23651602

[B16] Yanos P Moos R Determinants of functioning and well-being among individuals with schizophrenia: an integrated model Clin Psychol Rev 2007 27 1 58 77 10.1016/j.cpr.2005.12.008 16480804 PMC1790965

[B17] Dangelmaier RE Docherty NM Akamatsu TJ Psychosis proneness, coping, and perceptions of social support Am J Orthopsychiat 2006 76 1 13 17 16569121 10.1037/0002-9432.76.1.13

[B18] Fonseca-Pedrero E Lemos-Giraldez S Paino ME Sierra-Baigrie S Villazon-Garcia U Bobes J Muniz J Coping strategies in adolescents with psychotic-like experiences. [Conference Abstract] Europ Psychiat 2010 25 Supp 1 1592

[B19] Jalbrzikowski M Sugar CA Zinberg J Bachman P Cannon TD Bearden CE Coping styles of individuals at clinical high risk for developing psychosis Early Intervention Psychiat 2012 doi:10.1111/eip.12005 10.1111/eip.12005 PMC3582773 23164368

[B20] Lin A Wigman JTW Nelson B Vollebergh WAM van Os J Baksheev G Ryan J Raaijmakers QAW Thompson A Yung AR The relationship between coping and subclinical psychotic experiences in adolescents from the general population–a longitudinal study Psychol Med 2011 41 12 2535 2546 10.1017/S0033291711000560 21524327

[B21] Kaufman J Birmaher B Brent D Rao U Flynn C Moreci P Williamson D Ryan N Schedule for affective disorders and schizophrenia for school-age children-present and lifetime version (K-SADS-PL): initial reliability and validity data J Am Acad Child Psy 1997 36 7 980 988 10.1097/00004583-199707000-00021 9204677

[B22] Chambers WJ Puig-Antich J Hirsch M Paez P Ambrosini PJ Tabrizi MA Davies M The assessment of affective disorders in children and adolescents by semistructured interview: test-retest reliability of the schedule for affective disorders and schizophrenia for school-age children, present episode version Arch Gen Psychiat 1985 42 7 696 702 10.1001/archpsyc.1985.01790300064008 4015311

[B23] Shaffer D Gould MS Brasic J Ambrosini P Fisher P Bird H Aluwahlia S A children's global assessment scale (CGAS) Arch Gen Psychiat 1983 40 11 1228 1231 10.1001/archpsyc.1983.01790100074010 6639293

[B24] Frydenberg E Lewis R Boys play sport and girls turn to others: age, gender and ethnicity as determinants of coping J Adolescence 1993 16 3 253 266 10.1006/jado.1993.1024 8282897

[B25] O’Connor BP SPSS and SAS programs for determining the number of components using parallel analysis and Velicer’s MAP test Behav Res Methods 2000 32 3 396 402 10.3758/BF03200807 11029811

[B26] Kelleher I Devlin N Wigman JTW Kehoe A Murtagh A Fitzpatrick C Cannon M Psychotic experiences in a mental health clinic sample: implications for suicidality, multimorbidity and functioning Psychol Med 2013 1 10 http://dx.doi.org/10.1017/S0033291713002122 24025687 10.1017/S0033291713002122

[B27] Malla A Payne J First-episode psychosis: psychopathology, quality of life, and functional outcome Schizophr Bull 2005 31 3 650 671 10.1093/schbul/sbi031 16006593

[B28] Lin A Wood SJ Yung AR Measuring psychosocial outcome is good Curr Opin Psychiatr 2013 doi:10.1097/YCO.0b013e32835d82aa 10.1097/YCO.0b013e32835d82aa 23318660

[B29] Wardenaar KJ Wigman JTW Lin A Killackey E Collip D Wood SJ Ryan J Beksheev G Cosgrave E Nelson B Yung AY Development and validation of a new measure of everyday adolescent functioning: the multidimensional adolescent functioning scale J Adol Health 2013 52 195 200 10.1016/j.jadohealth.2012.06.021 23332484

[B30] Keshavan MS DeLisi LE Seidman LJ Early and broadly defined psychosis risk mental states Schizophr Res 2011 126 1–3 1 10 21123033 10.1016/j.schres.2010.10.006 PMC3388534

[B31] Lin A Wood SJ Nelson B Brewer WJ Spiliotacopoulos D Bruxner A Broussard C Pantelis C Yung AR Neurocognitive predictors of functional outcome two to 13 years after identification as ultra-high risk for psychosis Schizophr Res 2011 132 1 7 10.1016/j.schres.2011.06.014 21763109

[B32] Carrión RE McLaughlin A Goldberg TW Auther AM Olsen RH Olvet DM Correll CU Cornblatt BA Prediction of functional outcome in individuals at clinical high risk for psychosis JAMA Psychiatr 2013 70 11 1133 1142 10.1001/jamapsychiatry.2013.1909 PMC4469070 24006090

[B33] Morgan C Fisher H Environment and schizophrenia: environmental factors in schizophrenia: childhood trauma—a critical review Schizophr Bull 2007 33 1 3 10 17105965 10.1093/schbul/sbl053 PMC2632300

[B34] Varese F Smeets F Drukker M Lieverse R Lataster T Viechtbauer W Read J van Os J Bentall RP Childhood adversities increase the risk of psychosis: a meta-analysis of patient-control, prospective-and cross-sectional cohort studies Schizophr Bull 2012 38 4 661 671 10.1093/schbul/sbs050 22461484 PMC3406538

[B35] Kelleher I Harley M Lynch F Arseneault L Fitzpatrick C Cannon M Associations between childhood trauma, bullying and psychotic symptoms among a school-based adolescent sample Br J Psychiatr 2008 193 5 378 382 10.1192/bjp.bp.108.049536 18978317

[B36] Read J van Os J Morrison AP Ross CA Childhood trauma, psychosis and schizophrenia: a literature review with theoretical and clinical implications Acta Psychiat Scand 2005 112 5 330 350 10.1111/j.1600-0447.2005.00634.x 16223421

[B37] Docherty NM St-Hilaire A Aakre JM Seghers JP Life events and high-trait reactivity together predict psychotic symptom increases in schizophrenia Schizophr Bull 2009 35 3 638 645 10.1093/schbul/sbn002 18245057 PMC2669571

[B38] Tessner KD Mittal V Walker EF Longitudinal study of stressful life events and daily stressors among adolescents at high risk for psychotic disorders Schizophr Bull 2011 37 2 432 441 10.1093/schbul/sbp087 19734244 PMC3044629

[B39] Lataster J Myin-Germeys I Lieb R Wittchen HU van Os J Adversity and psychosis: a 10‒year prospective study investigating synergism between early and recent adversity in psychosis Acta Psychiat Scand 2011 125 5 388 399 22128839 10.1111/j.1600-0447.2011.01805.x

[B40] Horan WP Ventura J Nuechterlein KH Subotnik KL Hwang SS Mintz J Stressful life events in recent-onset schizophrenia: reduced frequencies and altered subjective appraisals Schizophr Res 2005 75 2 363 374 15885527 10.1016/j.schres.2004.07.019

[B41] Phillips LJ Edwards J McMurray N Francey S Comparison of experiences of stress and coping between young people at risk of psychosis and a non-clinical cohort Behav Cogn Psychother 2012 40 1 69 88 10.1017/S1352465811000397 21729339

[B42] Myin-Germeys I van Os J Stress-reactivity in psychosis: evidence for an affective pathway to psychosis Clin Psychol Rev 2007 27 4 409 424 10.1016/j.cpr.2006.09.005 17222489

[B43] Myin-Germeys I van Os J Schwartz JE Stone AA Delespaul PA Emotional reactivity to daily life stress in psychosis Arch Gen Psychiat 2001 58 12 1137 1144 10.1001/archpsyc.58.12.1137 11735842

[B44] Myin-Germeys I Krabbendam L Delespaul PA van Os J Do life events have their effect on psychosis by influencing the emotional reactivity to daily life stress? Psychol Med 2003 33 02 327 333 10.1017/S0033291702006785 12622311

[B45] Lataster T Wichers T Jacobs N Mengelers M Derom C van Thiery E Os J Myin-Germeys I Does reactivity to stress cosegregate with subclinical psychosis? A general population twin study Acta Psychiat Scand 2009 119 1 45 53 10.1111/j.1600-0447.2008.01263.x 18822092

[B46] Farhall J Greenwood KM Jackson HJ Coping with hallucinated voices in schizophrenia: a review of self-initiated strategies and therapeutic interventions Clin Psychol Rev 2007 27 4 476 493 10.1016/j.cpr.2006.12.002 17223238

[B47] Mortan O Sutcu PST Kose GG A pilot study on the effectiveness of a group-based cognitive-behavioral therapy program for coping with auditory hallucinations Turkish J Psychiat 2011 22 1 1 8 21360353

[B48] Sarin F Wallin F Widerlöv B Cognitive behavior therapy for schizophrenia: a meta-analytical review of randomized controlled trials Nordic J Psychiat 2011 65 3 162 174 10.3109/08039488.2011.577188 21563994

[B49] Compas BE Connor_smith JK Saltzman H THomson A Wadsworth ME Coping with stress during childhood and adolescence: problems, progress, and potential in theory and research Psychol Bull 2001 127 1 87 127 11271757

[B50] Coyne JC Downey G Social factors and psychopathology: stress, social support, and coping processes Ann Rev Psychol 1991 42 1 401 425 10.1146/annurev.ps.42.020191.002153 2018399

[B51] Grealish A Rai S Hunter A Morrison AP Qualitative exploration of empowerment from the perspective of young people with psychosis Clin Psychol Psychot 2013 20 2 136 148 10.1002/cpp.785 21882298

